# Progressive Learning Hill Climbing Algorithm with Energy-Map-Based Initialization for Image Reconstruction

**DOI:** 10.3390/biomimetics8020174

**Published:** 2023-04-22

**Authors:** Yuhui Zhang, Wenhong Wei, Zijia Wang

**Affiliations:** 1 School of Computer Science and Technology, Dongguan University of Technology, Dongguan 523808, China; 2 School of Computer Science and Cyber Engineering, Guangzhou University, Guangzhou 510006, China

**Keywords:** energy map, hill climbing, image reconstruction, metaheuristic, progressive learning strategy

## Abstract

Image reconstruction is an interesting yet challenging optimization problem that has several potential applications. The task is to reconstruct an image using a fixed number of transparent polygons. Traditional gradient-based algorithms cannot be applied to the problem since the optimization objective has no explicit expression and cannot be represented by computational graphs. Metaheuristic search algorithms are powerful optimization techniques for solving complex optimization problems, especially in the context of incomplete information or limited computational capability. In this paper, we developed a novel metaheuristic search algorithm named progressive learning hill climbing (ProHC) for image reconstruction. Instead of placing all the polygons on a blank canvas at once, ProHC starts from one polygon and gradually adds new polygons to the canvas until reaching the number limit. Furthermore, an energy-map-based initialization operator was designed to facilitate the generation of new solutions. To assess the performance of the proposed algorithm, we constructed a benchmark problem set containing four different types of images. The experimental results demonstrated that ProHC was able to produce visually pleasing reconstructions of the benchmark images. Moreover, the time consumed by ProHC was much shorter than that of the existing approach.

## 1. Introduction

Image reconstruction refers to the task of reconstructing an image with the restrictions of using specific geometric shapes, e.g., polygons or eclipses. It can be viewed as an optimization problem that minimizes the difference between the reconstructed image and the original image. Finding algorithms that can efficiently solve this problem may give rise to several important applications. One potential application is image compression. Instead of recording the pixel values, one can represent images with a relatively small number of polygons. Given the end points of the polygons and their corresponding colors and transparencies, one can repaint the image. Another potential application would be generating computational art works. Elaborate pictures can be created by using simple geometric shapes to approximate real-world figures.

The image reconstruction problem is very challenging since it comprises many decision variables, which are highly correlated. Moreover, there is no explicit expression for the objective function, making it infeasible to use gradient-based algorithms. To evaluate the fitness of a candidate solution, one needs to draw the polygons on a blank canvas and then calculate the element-wise differences between the reconstructed image and the source image. This evaluation process is very time-consuming. It is impractical for general computing devices to perform a large number of fitness evaluations. From this viewpoint, image reconstruction is a sort of expensive optimization problem [1,2,3,4,5].

Metaheuristic search algorithms are powerful optimization techniques that make no assumption about the nature of the problem and do not require any gradient information. Therefore, they can be applied to a variety of complex optimization problems, especially in the cases of imperfect information or a limited computational capability. According to the number of candidate solutions maintained in the search process, metaheuristic algorithms can be divided into two categories, i.e., single-solution and population-based algorithms. Single-solution-based algorithms iteratively update a single candidate solution so as to push the solution toward a local or global optimum. Some prominent single-solution algorithms include hill climbing [6,7,8], tabu search [9,10], and simulated annealing [11,12]. In contrast to single-solution algorithms, population-based algorithms maintain a population of candidate solutions in the running process. New solutions are generated by extracting and combing the information of several parental solutions. This type of algorithm can be further divided into two subgroups, i.e., evolutionary algorithms (EAs) and swarm intelligence (SI) algorithms. The design of these algorithms was inspired by the theory of evolution (e.g., genetic algorithms (GAs) [13,14,15,16], genetic programming (GP) [17,18,19,20,21], differential evolution (DE) [22,23,24,25,26], and evolutionary strategies (ESs) [27,28,29,30]) or the collective behavior of social animals (e.g., particle swarm optimization (PSO) [31,32,33,34,35] and ant colony optimization (ACO) [36,37,38,39,40,41]).

Since no gradient information is available, metaheuristic search algorithms have become one of the major tools for image reconstruction. In addition, due to the fact that evaluating the fitness of candidate solutions requires a large amount of computation, the running time may be unaffordable if population-based search algorithms are adopted. Therefore, single-solution-based algorithms have been widely used in the literature.

Hill climbing [8] is a popular single-solution-based algorithm. It starts with a random candidate solution and tries to improve the solution by making incremental changes. If a change improves the solution quality, then the change is kept and another incremental change is made to the solution. This process is repeated until no better solutions can be found or the algorithm reaches the predefined limit of fitness evaluations. Each incremental change is defined by random modifications to one or several elements of the candidate solution. This strategy can produce decent results for image reconstruction. However, it does not fully resolve the challenges that arise in the optimization process. Note that each candidate solution is composed of multiple geometric shapes, and all the geometric shapes together form the reconstructed image. The decision variables that determine the position, color, and transparency of the geometric shapes highly correlate with each other. A small change in one geometric shape may influence the appearance of other shapes that overlap with it. On the other hand, the total number of decision variables is several times the number of geometric shapes used to approximate the source image. As the number of shapes increases, the number of decision variables increases rapidly. This causes the rapid growth of the search space, which significantly lowers the search efficiency of hill climbing.

To overcome the challenges posed by the image reconstruction problem, researchers have drawn inspiration from approaches in mathematical optimization and deep learning. In mathematical optimization, it is an effective strategy to divide a complex optimization problem into a sequence of simple problems. Optimizing a sequence of problems one after another provides an approximate solution to the original complex problem [41]. This strategy also applies to many tasks in deep learning. Karras et al. [42] developed a new training methodology for generative adversarial networks (GANs) to produce high-resolution images. The key idea is to grow both generator and discriminator networks progressively. Starting from low-resolution images, the methodology adds new layers that introduced higher-resolution details as the training progresses. The detailed architecture of this methodology can be found in [42]. Its incremental learning nature allows GANs to first discover the large-scale structure of image distribution and then shift attention to finer-scale structures, instead of learning all the structures simultaneously. The experimental results showed that the new methodology was able to speed up and stabilize the training process. Tatarchenko et al. [43] developed a deep convolution decoder architecture called the octree generating network (OGN) that is able to generate volumetric 3D outputs. OGN represents its output as an octree. It gradually refines the initial low-resolution structure to higher resolutions as the network goes deeper. Only a sparse set of spatial locations is predicted at each level. More detailed information about the structure of the OGN can be found in [43]. The experimental results proved that OGN had the ability to produce high-resolution outputs in a computation- and memory-efficient manner.

From the above review, it can be noticed that progressively increasing the complexity of learning tasks is an effective strategy for various applications. In addition, this strategy is able to increase the learning speed. Motivated by this finding, we developed a progressive learning strategy for the hill climbing algorithm so that it could progressively find a way of combining basic geometric shapes to approximate different types of images. The basic idea was to reconstruct images from a single geometric shape. After optimizing the variables of the first shape, we stacked a new shape on top of the existing ones. The process was repeated until we reached the number limit. This process amounted to transforming the original complex high-dimensional problem into a sequence of simple lower-dimensional problems. In the problem sequence, the optimization outcome of the former problem served as the starting point of the latter problem. In this way, the challenges created by a high-dimensional search space and variable correlation could be addressed. In addition, an energy-map-based mutation operator was incorporated into our algorithm to facilitate the generation of promising new solutions, which further enhanced the search efficiency.

To evaluate the performance of the proposed algorithm, we constructed a benchmark problem set that contained four different types of images. Experiments were conducted on the benchmark problem set to check the efficacy of the proposed progressive learning strategy, as well as the energy-map-based initialization operator. The experimental results demonstrated that the new strategies were able to enhance the optimization performance and generate images of a higher quality. In addition, the running time was significantly reduced.

The remainder of this paper is organized as follows. Section 2 provides a formal description of the image reconstruction problem. Then, the existing hill climbing algorithm is introduced in detail. Section 3 introduces related studies in the literature. The proposed progressive learning strategy and energy-map-based initialization operator are described in Section 4, where we present the underlying principle of the new strategies. In Section 5, we describe experiments conducted on a set of benchmark images to evaluate the effectiveness of the proposed strategies, with a detailed analysis of the numerical results. Finally, conclusions are drawn in Section 6. Some promising future research directions are pointed out as well.

## 2. Background

In this section, a formal description of image reconstruction is first presented. Then, we review the classical single-solution algorithm to lay the groundwork for the proposed progressive learning strategy presented in Section 4. We also discuss some recent studies closely related to the topic of image reconstruction.

### 2.1. Image Reconstruction Problem

**Figure 1:**
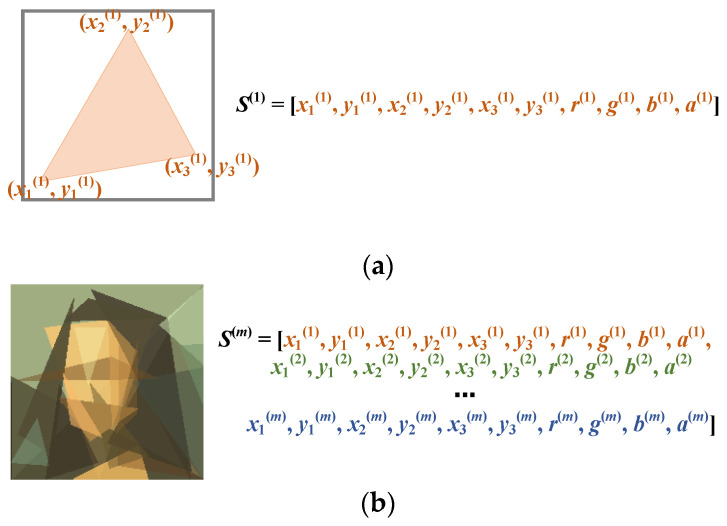
Encoding of a candidate solution to the image reconstruction problem. (**a**) Candidate solution consisting of a single polygon (triangle), (**b**) candidate solution consisting of *m* polygons (triangles).

It is interesting to note that the image reconstruction problem was first posed in a blog published by Roger Johansson [44]. Johansson presented the idea of reconstructing one of the most famous paintings in the world, the Mona Lisa, using a number of transparent polygons. One would need to adjust the shape and position of the polygons, as well as their colors and transparency, so as to make the reconstructed image as close to the original image as possible. This task can be formulated as an optimization problem, in which the decision variables correspond to the parameters of the polygons. Suppose that the *i*-th polygon *P_i_* has *n_i_* vertices; then, *P_i_* can be represented by a list of parameters, i.e., [*x*_1_, *y*_1_, *x*_2_, *y*_2_, …, *x_ni_*, *y_ni_*, *r*, *g*, *b*, *a*]. The tuple (*x_j_*, *y_j_*) denotes the coordinate of the *j*-th vertex. The last four elements encode the color and transparency of *P_i_*. Suppose there are, overall, *m* polygons; a candidate solution is represented by a sequence of polygons, i.e., [*P*_1_, *P*_2_, …, *P_m_*]. If all the polygons have the same number of vertices, i.e., *n*, then the total number of decision variables would be (2*n* + 4) × *m*. Therefore, the number of decision variables grows linearly as the number of polygons increases. Figure 1 illustrates the solution encoding. In the figure, each polygon has three vertices. The parameters of the polygons are concatenated to form a long vector.

Given a candidate solution containing *m* polygons, the reconstructed image ***Y*** can be expressed as:

(1) $$Y=f([P_{1},P_{2},\ldots ,P_{m}]),$$

where *f* is the reconstruction function. This is generally implemented using off-the-shelf graphics interfaces. One thing worth noting is that the reconstruction function *f* has no explicit expression. It is also very difficult to build a computational graph for this function.

**Figure 2:**
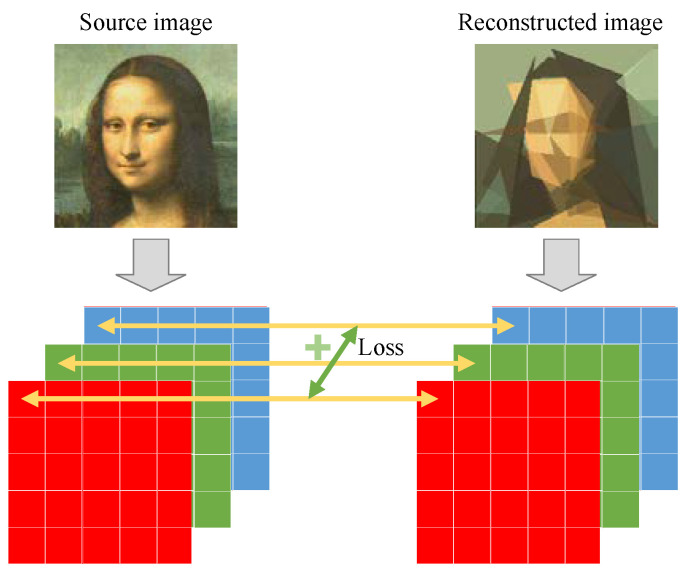
Illustration of objective function computation. The sum of element-wise absolute differences is used as the loss function.

The sum of the element-wise absolute difference between the source image ***X*** and the reconstructed image ***Y*** is used as the objective function, as illustrated in Figure 2. Suppose that the size of source image ***X*** is *W* × *H* × *C*; the objective can be formulated as follows:

(2) $$Loss(X,Y)=\sum_{i=1}^{W}\sum_{j=1}^{H}\sum_{k=1}^{C}\left|X_{i,j,k}-Y_{i,j,k}\right|,$$

where *W*, *H,* and *C* denote the width, height, and number of channels of the image. A channel is the grayscale image of a colored image, which is made up of only one of the primary colors that form the colored image. Primary colors are basic colors that can be mixed together to produce other colors. For RGB images, the primary colors are red, green, and blue. Each pixel of an RGB image is made up of three channels, with each channel representing a primary color. The goal is to minimize the objective function. A zero-function value indicates that the polygons completely restore the source image ***X***.

The image reconstruction problem has several potential applications. The most direct application is image compression. One can store an image as the parameters of the polygons instead of the pixel values. Less memory is required, since the number of parameters is much smaller than the number of pixel values. The second application is generating artistic images. Any complex figure can be represented by simple geometric shapes. The level of abstraction can be adjusted by controlling the number of polygons used.
**Algorithm 1** Hill climbing for image reconstruction**Input**: Source image ***X***, number of polygons *m*, number of vertices in each polygon *n*.**Output**: A sequence of polygons [*P*_1_, *P*_2_, …, *P_m_*] with specified parameter values.1: Generate an initialized solution *S*_0_ by randomly sampling the parameter values from the search range.2: Calculate the objective function value *L*_0_ of the initial solution *S*_0_.3: *t* = 04: **while**
*t < MaxFEs*:5:   *S_t_*_+1_←*S_t_*, *I* ← *rand_int*(*m*)6:   *r*_1_
***=***
*rand*(0, 3)7:   **if** *r*_1_ < 1 **then:**8:      Mutate_color(*S_t_*_+1_.*P_i_*)9:   **else if**
*r*_1_ < 2 **then:**10:      Mutate_vertex(*S_t_*_+1_.*P_i_*)11:   **else**: // Mutate the polygon sequence.12:      Mutate_sequence(*S_t_*_+1_, *i*)13:   **end if**14:   Calculate the objective function value *L_t_*_+1_ of the new solution *S_t_*_+1_.15:   **if**
*L_t_*_+1_ < *L_t_*
**then:**16:      *S_t_*_+1_ ← *S_t_*;17:   **end if**18:   *t*++;19: **end while**

### 2.2. Hill Climbing with Mutation Operators

The objective function calculates the difference between the source image ***X*** and the reconstructed image ***Y***. However, to reconstruct images from the parameters of polygons, one needs to use the reconstruction function *f*, which has no explicit expression. Consequently, the objective function cannot be expressed as a function of the parameters of polygons. This property limits the application of gradient-based methods [45]. In comparison, hill climbing [8] is an iterative optimization technique that does not require any gradient information. It can be used for solving any black-box optimization problem in which the objective function or the system being optimized is treated as a black box. The simplicity of hill climbing makes it popular among researchers. Hill climbing has been adopted to handle the image reconstruction problem. The pseudo code is presented in Algorithm 1 [44].
**Algorithm 2** Mutate_color**Input**: A polygon *P_i_*.**Output**: Mutated *P_i_*. 1: *r*_1_
***=***
*rand*(0, 1)2: *r*_2_
***=***
*rand*(0, 1)3: **if** *r*_1_
*<* 1/4 **then**:4:    **if**
*r*_2_
*<* 0.5 **then:**5:        *P_i_.a* ← *S_t_*_+1_.*P_i_.a* + *rand*(0.1)6:    **else:**7:        *P_i_.a* ← *rand*(0, 1)8:    **end if**9: **else if** *r*_1_
*<* 2/4 **then**:10:    **if**
*r*_2_
*<* 0.5 **then:**11:        *P_i_.r* ← *S_t_*_+1_.*P_i_.r* + *rand*()12:    **else:**13:        *P_i_.r* ← *rand*(0, 1)14:    **end if**15: **else if** *r*_1_
*<* 3/4 **then**:16:    **if**
*r*_2_
*<* 0.5 **then:**17:        *P_i_.g* ← *S_t_*_+1_.*P_i_.g* + *rand*()18:    **else:**19:        *P_i_.g* ← *rand*(0, 1)20:    **end if**21: **else:**22:    **if**
*r*_2_
*<* 0.5 **then:**23:        *P_i_.b* ← *S_t_*_+1_.*P_i_.b* + *rand*()24:    **else:**25        *P_i_.b* ← *rand*(0, 1)26:    **end if**27: **end if**

**Figure 3:**
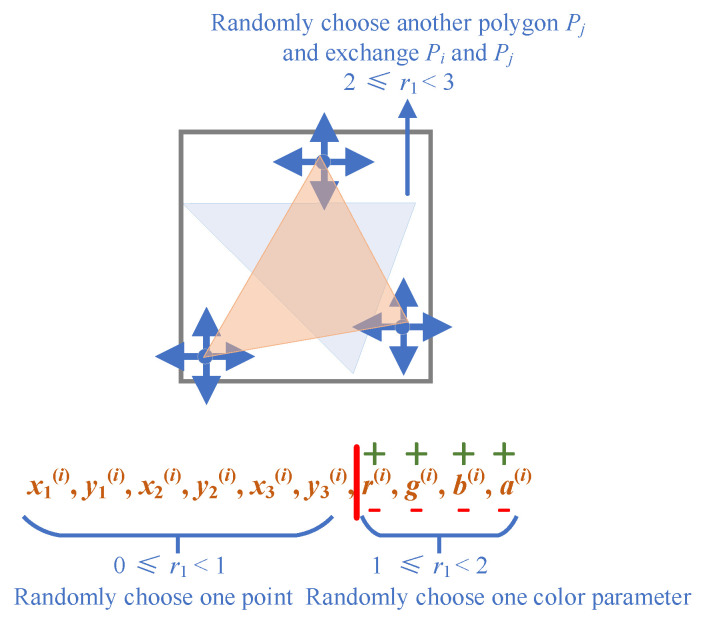
Illustration of the mutation operator used in the hill climbing algorithm.

Hill climbing starts with a random candidate solution *S*_0_, in which all the parameters of the polygons are randomly sampled from their feasible regions. Then, it enters a loop that tries to iteratively improve the candidate solution. Suppose that the current candidate solution is *S_t_*; a mutation operator that makes random changes to *S_t_* is designed to produce new solutions. Specifically, a polygon from the candidate solution is randomly chosen, and one of its parameters is changed. Suppose that the *i*-th polygon *P_i_* is chosen to be modified. A random real number *r*_1_ within [0.0, 3.0] is first generated. If *r*_1_ is less than one, then the color or transparency of the polygon is changed (Algorithm 2). The parameters *a*, *r*, *g*, and *b* have an equal chance of being modified. If *r*_1_ is in the range [1.0, 2.0], the position of a vertex is modified (Algorithm 3). The value of the *x*-coordinate or the *y*-coordinate is changed. In the above two cases, the selected parameter value is either increased by a small value or set to a random value within its feasible range. The decision is made by sampling another random value *r*_2_ within [0.0, 1.0]. If *r*_1_ is larger than two, another polygon *P_j_* is randomly selected, and the stacking sequence of *P_i_* and *P_j_* is exchanged (Algorithm 4). A new solution *S_t_*_+1_ is generated in this manner. Subsequently, the objective function value *L_t_*_+1_ of the new solution is calculated. If its objective function value *L_t_*_+1_ is less than that of *S_t_*, then *S_t_*_+1_ is replaced with *S_t_*. Otherwise, the new solution *S_t_*_+1_ enters the next iteration. The above process is repeated until the maximum number of iterations is reached. Figure 3 illustrates the mutation operator. For a randomly selected polygon *P_i_*, the mutation operator makes changes to one of three components, namely, the position of the vertices, the color/transparency, and the position in the stacking sequence.
**Algorithm 3** Mutate_vertex**Input**: A polygon *P_i_*.**Output**: Mutated *P_i_*. 1: *j* ← *rand_int*(*n*)2: *r*_1_
***=***
*rand*(0, 1), *r*_2_
***=***
*rand*(0, 1)3: **if** *r*_1_ < 0.5 **then**:4:    **if**
*r*_2_
*<* 0.5 **then:**5:        *P_i_.x_j_* = *S_t_*_+1_.*P_i_.x_j_* + *rand*(0, 0.1 *W*)6:    **else**:7:        *P_i_.x_j_* = *rand*(0, *W*)8:    **end if**9: **else**:10:    **if**
*r*_2_
*<* 0.5 **then:**11:        *P_i_.y_j_* = *S_t_*_+1_.*P_i_.y_j_* + *rand*(0, 0.1 *H*)12:    **else**:13:        *P_i_.y_j_* = *rand*(0, *H*)14:    **end if**15: **end if**

**Algorithm 4** Mutate sequence**Input**: A sequence of polygons *S_t_*_+1_, selected index *i*. **Output**: Mutated sequence.1: *j* ← *rand_int*(*m*)2: *tmp* ← *S_t_*_+1_.*P_i_*3: *S_t_*_+1_.*P_i_*← *S_t_*_+1_.*P_j_*4: *S_t_*_+1_.*P_j_* ← *tmp*

Because a mutation operator is used in the algorithm to generate new solutions, its developer called the algorithm GP instead of hill climbing. However, since only one candidate solution is maintained in the search process, and there are no interactions between the generated solutions, it would be more appropriate to name it hill climbing.

## 3. Related Studies

Before Johansson posed the image reconstruction problem, researchers had examined similar problems. Castro et al. [46] proposed a clone selection algorithm (CLONALG) that minimized the affinity maturation of the immune response. The principle was that only cells that recognized the antigens were selected to proliferate. The selected cells underwent a maturation process that improved their affinity to the antigens. The authors applied the algorithm to perform machine learning and pattern-recognition tasks. One of the tasks they tested was to restore grey number images from random pixel values. As the search progressed, the candidate solutions in the memory cell gradually approached the original images. For a demonstration of the optimization process, interested readers can refer to [46]. This problem can be regarded as a special case of the image reconstruction problem, where the polygons are replaced by pixels. Since the goal is to approximate small-scale grey images, the number of parameters to be optimized is much smaller than that of the image reconstruction problem.

Tian and Ha [47] revisited the use of EAs for computational creativity and developed an evolution strategy (ES) for generating art images. The ES was used to fit both concrete images and abstract concepts. The task of fitting concrete images is the same as image reconstruction, in which the objective function is defined as *L*_2_, i.e., the loss between the generated image and the source image. Fitting abstract concepts is less straightforward than fitting concrete images. An abstract concept was given in the form of a text prompt. The match degree of the generated image and the given text prompt was measured using a transferable visual model called CLIP. CLIP jointly trained a text encoder and an image encoder to predict the correct pairing of texts and images. The text prompt and the generated image were fed into the text encoder and the image encoder, respectively. The objective function was defined as the cosine distance between the output of the text encoder and the output of the image encoder. The ES was used to adjust the parameters of the polygons with the aim of minimizing the cosine distance. As the evolution progressed, the generated image gradually matched the abstract concept indicated by the given text prompt. Interested readers can refer to [47] for some interesting examples of abstract concept fitting.

## 4. Progressive Learning Hill Climbing Algorithm with an Energy-Map-Based Initialization Operator

In this section, we first illustrate the idea behind the progressive learning strategy and explain how the strategy can be used to circumvent the challenges posed by the image reconstruction problem. We developed a new algorithm named ProHC by combining the progressive learning strategy with the hill climbing algorithm. Furthermore, to improve the search efficiency, an energy-map-based initialization operator was designed to better adjust the parameters of the polygons.

### 4.1. Progressive Learning Strategy

The image reconstruction problem is very challenging since it involves a large number of decision variables. In addition, the variables are highly correlated. Slight changes in the parameters of one polygon affect the appearance of other polygons stacked in the same position. Although hill climbing is able to generate quite appealing results for this problem, there is still large room for improvement. The search efficiency can be further enhanced if the challenges can be overcome. Motivated by the successful application of the progressive learning concept in mathematical optimization and deep learning areas [41,42,43], we developed a progressive learning strategy for solving the image reconstruction problem.

**Figure 4:**
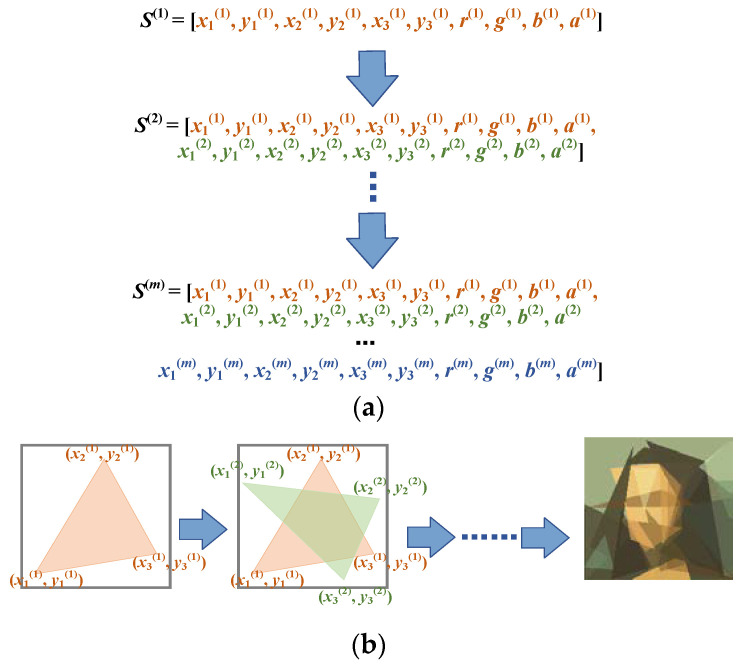
Illustration of progressive learning strategy. Starting from a single polygon, new polygons are added to the canvas after the parameters of the previous polygons have been sufficiently optimized. (**a**) Genotype viewpoint: progressively increasing the number of parameters; (**b**) phenotype viewpoint: progressively stacking new polygons on the canvas.

The basic idea of the progressive learning strategy is very simple. Instead of simultaneously optimizing the parameters of all the polygons, we adjusted the color, transparency, and vertices of the polygons sequentially. A newly generated polygon was stacked on top of the previous polygons after the parameters of the polygons were sufficiently optimized. This process amounted to transforming the original complex problem into a sequence of simpler problems. Starting from a single polygon, we gradually increased the number of decision variables by adding new polygons on top of the existing ones. The last problem in the problem sequence was the same as the original problem. This guaranteed that solutions to any problem in the problem sequence were partial solutions to the original problem. The progressive learning strategy is illustrated in Figure 4 from both genotype and phenotype viewpoints.

The pseudo-code of the hill climbing algorithm with the progressive learning strategy is presented in Algorithm 5. In contrast to traditional hill climbing, ProHC stacks the polygons layer by layer until the number of polygons reaches the predefined limit. The polygons in different layers have different probabilities of mutation. Polygons in newer layers have higher probabilities. Specifically, the selection probabilities are assigned as follows. In the initial phase, there is only one polygon. All the attention is focused on the optimization of the first polygon. When the objective function value has not been improved for a number of successive trials, one should check whether the inclusion of the previously added polygon contributed to a reduction in the objective function value. If the condition is false, then the previously added polygon should be reinitialized. Otherwise, a new polygon is added to the canvas. In subsequent iterations, half of the mutation probability is assigned to the new polygon. The remaining probability is assigned to the preceding polygons according to a geometric sequence. Every time a new polygon is added, ProHC reassigns the probabilities in the same manner. Finally, after the number of polygons reaches the predefined number limit, all the polygons are assigned equal probabilities of mutation. In this phase, the problem becomes the same as the original problem. Through the progressive learning process, a high-quality initial solution is obtained. The remaining computational resources (objective function evaluations) are used to fine-tune the parameters of the polygons.
**Algorithm 5** Progressive learning hill climbing for image reconstruction**Input**: Source image ***X***, number of polygons *m*, number of vertices in each polygon *n*.**Output**: A sequence of polygons [*P*_1_, *P*_2_, …, *P_m_*] with specified parameter values.1: Generate an initialized solution *S*_0_ with a single polygon by randomly sample the parameter values from the search range2: Calculate the objective function value *L*_0_ of the initial solution *S*_0_3: Compute the energy map ***E***_0_4: *t* = 0; *k* ← 1, *V_k_* ← *L*_0_; *Cnt* ← 0 // stagnation counter5: [*pr*_1_] ← [1]6: **while**
*t < MaxFEs*:7:   *S_t_*_+1_←*S_t_*8:   *i* ← *rand_int*(*k*, [*pr_k_*_,_,…, *pr*_1_])9:   *r*_1_
***=***
*rand*(0, 3)10:   **if** *r*_1_ < 1 **then:**11:      Mutate_color(*S_t_*_+1_.*P_i_*)12:   **else if**
*r*_1_ < 2 **then:**13:      Mutate_vertex(*S_t_*_+1_.*P_i_*)14:   **end if**15:   Calculate the objective function value *L_t_*_+1_ of the new solution *S_t_*_+1_16:   **if**
*L_t_*_+1_ < *L_t_*
**then:**17:      *S_t_*_+1_ ← *S_t_*, *Cnt* ← 018:   **else:**19:      *Cnt* ← *Cnt* + 1;20:   **end if**21:   *t* ← *t* + 1;22:   **if** *Cnt* > *limit* and *k* < *m* **then**:23:      **if** *L_t_*_+1_ < *V_k_* **then**:24:         Update the energy map ***E***25:         Generate a random polygon *P_k+_*_1_26:         Energy_map_based_initialization(*P_k+_*_1_)27:         S*_t_*_+1_ ← [*S_t_*_+1_, *P_k+_*_1_]28:         [*pr_k_*_+1_, *pr_k_*, …, *pr*_1_] ← normalize([2^−1^, 2^−2^, …, 2^−*k*^, 2^−(*k*+1)^]) 29:         *V_k_* ← *L_t_*_+1_; *k* = *k*+1, *Cnt* ← 030:      **else:**31:      Energy_map_based_initialization(*S_t_*_+1_.*P_k_*)32:      *Cnt* ← *Cnt* + 133:      **end if**34:   **end if**35:   **if** *Cnt* > *limit* and *k* = *m* **then:**36:        [*pr_m_*, *pr_m_*_−1_, …, *pr*_1_] ← [1/*m*, 1/*m*, …, 1/*m*]37:   **end if**38: **end while**

**Figure 5:**
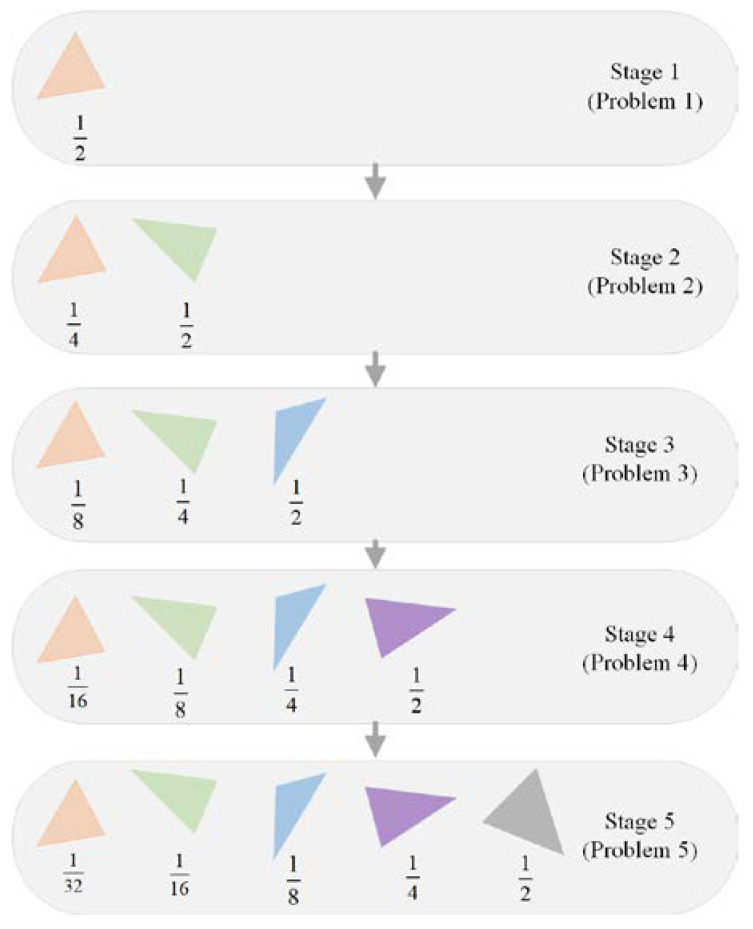
Geometric probability assignment strategy.

Figure 5 illustrates the probability assignment process using an example with five stages. When solving the *i*-th problem, the parameters of the previous *i* − 1 polygons are inherited from the solution of the (*i* − 1)th problem. These parameters have been optimized for a relatively large amount of time. In comparison, the parameters of the *i*-th polygon are new to the algorithm. Therefore, a larger amount of effort is spent on the new parameters by assigning a higher mutation probability to the *i*-th polygon. For the parameters of the previous *i* − 1 polygons, it can be inferred that the smaller the polygon number, the longer the time spent on its parameters. In order to make sure that all the parameters have similar chances of mutation, the probability assigned to each polygon is determined based on a geometric sequence. The probabilities in each stage are normalized so that they sum to one. In this way, the total mutation probability of each polygon across multiple stages is roughly the same.

The progressive learning strategy has two advantages. First, it can circumvent the challenges posed by the image reconstruction problem. In the problem sequence, solutions to the former problem can serve as partial solutions to later problems. Therefore, relatively good starting points can be obtained for the later problems by first optimizing the former problems. The former problems involve only a small number of polygons and have less control parameters. They are much easier to solve. Solving the problems one after another provides a high-quality initial solution for the original complex problem and makes the optimization process much easier. The second advantage is that the progressive learning strategy can reduce the evaluation time of the objective function. To calculate the objective function value of a candidate solution, one needs to draw the polygons on a blank canvas and then compute the element-wise differences between the generated image and the source image. Since the problems in the problem sequence encode less polygons than the original problem, it is less time-consuming to compute the reconstruction function *f*, and therefore the total running time of ProHC can be significantly reduced.

One thing worth noting is that ProHC does not incorporate an exclusion operator. The purpose of the exclusion operation is to remove redundant polygons. ProHC-EM has a similar mechanism that plays the role of the exclusion operator. Suppose that the final solution to the (*i* − 1)th problem is *S_i_*_−1_, and its objective function value is *f_i_*_−1_. One moves forward to the *i*-th problem by stacking the *i*-th polygon on top of the existing ones. After a period of optimization, one finds a solution *S_i_* to the *i*-th problem. If the objective function value of *S_i_* is worse than *f_i_*_−1_, the *i*-th polygon is reinitialized, and the optimization process is repeated. In this way, one can ensure that each newly added polygon is not redundant and contributes to the improvement of the objective function value.

### 4.2. Initialization Assisted by an Energy Map

In the progressive learning strategy, each time a new polygon is appended to the canvas, the parameters of the polygon are randomly sampled from their feasible regions. However, there exist more efficient ways to initialize the parameters. Since the goal of image reconstruction is to generate an image that matches the source image, it is reasonable to place new polygons on regions where the differences between the generated image and the source image are significant. When determining the vertices of the newly generated polygons, higher probabilities can be directed to positions with large biases.

To this end, we needed to construct a matrix that recorded the sum of element-wise absolute differences across the channel dimension between the generated image and the source image. The matrix is referred to as an energy map. High energies correspond to high differences. The energy map provides useful information about which part of the generated image is dissimilar to the source image. One can use this information to guide the initialization of the newly added polygons. Motivated by this finding, we developed an energy-map-based initialization operator. Every time a new polygon is added to the canvas, the operator is adopted to initialize the vertices of the polygon.
**Algorithm 6** Energy-map-based initialization**Input**: A polygon *P_i_*, energy map ***E***.**Output**: Initialized *P_i_*. 1: *j* ← *rand_int*(*n*)2: *P_i_.r* ← *rand*(), *P_i_.g* ← *rand*(), *P_i_.b*← *rand*(), *P_i_.a* ← *rand*()3: Compute the probability matrix ***Pr***4: Compute the supplemental matrix ***MX***5: **for** *k* = 1, …, *n*:6:   *r*_1_
***=***
*rand*(0, 1)7:   Find the first element (*I*, *j*) in matrix ***MX*** that has a larger value than *r*_1_8:   *P_i_.*(*x_k_*, *y_k_*) ← (*i*, *j*)9: **end for**

The pseudo-code of the energy-map-assisted initialization operator is presented in Algorithm 6. Instead of randomly initializing the positions of the vertices, probabilities are assigned to pixels with respect to their corresponding energies. Specifically, the probability of selecting position (*i*, *j*) as a vertex of the polygon is calculated as follows:

(3) $$Pr_{i,j}=\frac{E_{i,j}}{\sum_{i=1}^{W}\sum_{j=1}^{H}E_{i,j}},$$

where *E_i_*_,*j*_ denotes the energy associated with pixel (*i*, *j*), which is defined as follows:

(4) $$E_{i,j}=\sum_{k=1}^{C}\left|X_{i,j,k}-Y_{i,j,k}\right|.$$

To sample the vertices of the new polygon, a supplemental matrix ***MX*** is first computed. The elements of ***MX*** are the cumulated probabilities of matrix ***Pr***. Specifically, the element *mx_i_*_,*j*_ in position (*i*, *j*) is computed as follows:

(5) $$mx_{i,j}=\sum_{k=1}^{i-1}\sum_{l=1}^{H}Pr_{k,l}+\sum_{l=1}^{j}Pr_{i,l}.$$

When sampling a new vertex, a random real value *r* whin [0, 1] is generated. Then, one retrieves the first element *mx_i_*_,*j*_ in matrix ***MX*** whose value is larger than *r*. The coordinate (*i*, *j*) is selected as the position of the new vertex. All vertices of the new polygon are determined in the same manner. In this way, there is a higher probability that the new polygon is placed on the most critical regions. With the energy-map-based operator, ProHC-EM (ProHC with an energy map) can avoid wasting effort on low-energy regions and further increase the search efficiency. The proposed approach relates to bionics in two ways:

- HC is a useful tool in bionics for optimizing the performance of artificial systems inspired by biological systems. The proposed progressive learning strategy can be embedded into HC to further improve its effectiveness. By mimicking the problem-solving procedures of human beings, ProHC can generate effective solutions to complex design problems, allowing researchers to create artificial systems that are more similar to biological systems in their structure, function, and behavior.
- ProHC-EM incorporates a mutation operator that mimics the process of evolution that occurs in biological systems. An incremental change is made to the candidate solution by mutating position-related or color-related parameters of a selected polygon. Moreover, an energy-map-based initialization operator was designed to help the algorithm target the most critical regions of the canvas and place new polygons on these regions. The effect of the energy map is similar to the heat-sensing pits of snakes in biology. The heat-sensing pits allow snakes to target prey by detecting the infrared radiation of warm-blooded animals.

### 4.3. Complexity Analysis

The proposed algorithm (shown in Algorithm 5) contains six major steps, namely, initialization, mutation, replacement, energy map update, and polygon increment. The initialization procedure (lines 1–5) runs in *O*(*mn + HW*) time. It is only executed once at the beginning of the algorithm. The other procedures are in the main loop of ProHC-EM and are executed repeatedly. Both mutation (lines 8–14) and replacement (lines 15–20) procedures consume a constant time. The time spent on the energy map update (line 24) is *O*(*HW*). The procedure used to determine whether to add a new polygon (lines 21–34) requires *O*(*HW*) time. The energy-map-based initialization is the most time-consuming step that dominates the other terms. According to the pseudo-code provided in Algorithm 6, the running time of the procedure is *O(nHW)*. Therefore, the overall time complexity of ProHC-EM is *O*(*nHW*) per iteration. In cases where *n* is set to small integers, the integration of the proposed progressive learning strategy and the energy-map-based initialization do not impose a serious burden on the complexity.

## 5. Experimental Study

In this section, we present experiments carried out to study the performance of the hill climbing algorithm developed based on the progressive learning strategy. We first constructed a set of benchmark test cases that contained different types of images. Then, the performance of ProHC-EM was evaluated in terms of the final objective function value and running time. The effect of the progressive learning strategy, as well as the energy-map-based initialization operator, are investigated in this section.

### 5.1. Experimental Setup

#### 5.1.1. Test Cases

**Figure 6:**
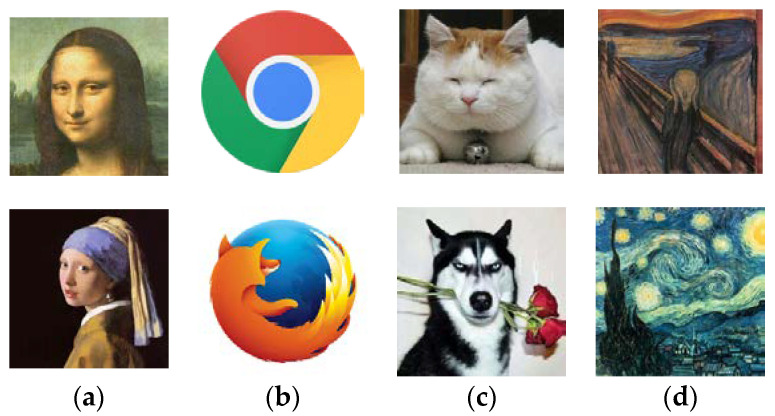
Benchmark test cases. (**a**) First category: famous portrait paintings; (**b**) second category: logos of popular browsers; (**c**) third category: pictures of animals; (**d**) forth category: impressionist paintings.

A set of test cases with different characteristics were collected to examine the algorithm’s ability to reconstruct images. The benchmark set contained eight 200 × 200 images. They were divided into four categories. The first category contained two famous portrait paintings, i.e., the ‘Mona Lisa’ and the ‘Girl with a Pearl Earring’. The second category consisted of the logos of two popular web browsers (Chrome and Firefox). The images in the third category were photos of animals (i.e., a cat and a dog). The last category consisted of two famous artworks from impressionist artists, namely, ‘The Starry Night’ and ‘The Scream’. Figure 6 depicts the test cases. It can be observed that the second category was the easiest among the four categories, since the images involved a small number of solid colors, and the subjects were composed of simple geometric shapes. The last category was more difficult to reconstruct due to the features of impressionist paintings. In impressionist paintings, the essence of subjects is captured by short, thick strokes of paint. The artists produce greys and dark tones by mixing complementary colors.

#### 5.1.2. Performance Metric

To evaluate the quality of the reconstructed image, a performance metric that revealed the similarity between the reconstructed image and the source image needed to be specified. In the experiment, the complete percentage (*CP*) [44] was used as the performance metric. Specifically, this metric is defined as follows:

(6) $$CP=\frac{MaxL-L_{best}}{MaxL}\times 100,$$

where *L_best_* is the loss of the current best solution found, and *MaxL* is the difference between the source image and the blank canvas. This is computed as follows:

(7) MaxL=Loss(X,B),

where ***X*** is the source image, and ***B*** is the blank canvas whose pixel values equal zero. The larger the complete percentage, the better the performance of the reconstruction algorithm. Note that it was feasible to directly use the sum of element-wise absolute differences (i.e., *L_best_*) to evaluate the algorithm performance. However, the loss functions had different ranges for different test cases. Therefore, it was difficult to interpret the loss function value. In comparison, the complete percentage indicated how well the reconstructed image matched the source image. A 100% *CP* value would indicate that the reconstructed image was exactly the same as the source image, while 0% would indicate that the reconstructed image was a blank canvas.

### 5.2. Overall Performance

To show the overall effect of the proposed progressive learning strategy and the energy-map-based initialization operator, we compared ProHC-EM with the mutation-based hill climbing algorithm (denoted as HC) [44]. The termination criterion of the two algorithms was defined by the maximum number of fitness evaluations (*MaxFEs*). Specifically, *MaxFEs* was set to 5 × 10^5^ for all eight test cases. For each test case, 50 polygons were used to approximate the source image, and each polygon had three vertices, namely, *m* = 50 and *n* = 3. In the progressive learning strategy, the threshold value used to determine whether to add a new polygon was empirically set to 1000. To obtain statistically reliable results, both algorithms ran 25 times for each test case. The hyperparameters of ProHC-EM were set based on the following considerations:

- The hyperparameter *n* determined the number of end points of each polygon. Considering that triangles are the simplest type of polygon and can be used as building blocks for more complex polygons, *n* was set to 3.
- The hyperparameter *m* controlled the number of polygons used to reconstruct an image. Generally speaking, the larger the *m* value, the higher the precision of the reconstructed image. However, setting *m* to a large number would significantly increase the optimization time. The goal of the experimental study was to examine the effectiveness and robustness of the new strategies. Therefore, we made a compromise by setting *m* to 50.
- The hyperparameter *MaxFEs* was used to determine the termination criterion of the optimization algorithms. When *m* was set to 50 and *n* was set to 3, there were 500 decision variables in total. The hyperparameter *MaxFEs* was set to 1000 times the number of decision variables.

The experimental results of the algorithms for all eight test cases are listed in Table 1, with better results marked in bold. Table 1 provides the average *CP* values over 25 independent runs as well as the standard deviations. Moreover, we conducted a Wilcoxon sign rank test to examine the differences between the numerical results of the two algorithms. The *p*-values of the statistical tests are also included in Table 1. From the table, it can be observed that ProHC-EM consistently outperformed HC in all the test cases. For each test case, ProHC-EM surpassed HC by approximately one percent. It is worth noting that increasing the *CP* value in the late optimization stage was much harder than in the early optimization stage. This was because the high-energy region was gradually divided into many small parts as the optimization progressed. To reduce the energy in these small parts, we needed to fine-tune the parameters of the deployed polygons. This required a large number of fitness evaluations. From this point of view, the improvements brought by the progressive learning strategy and energy-map-based initialization were significant. Another observation was that ProHC-EM had lower standard deviations, indicating that ProHC-EM had more stable performance than HC. According to the *p*-values listed in Table 1, the *CP* values obtained by ProHC-EM were statistically different from those of HC at a significance level of *α* = 0.05. It is worth pointing out that the average *CP* values obtained by ProHC-EM for the second category were the highest among the four categories, while the numerical results for the fourth category were the lowest. This matched our intuition that the second category of images would be the simplest to reconstruct and the fourth category contained the most difficult test cases.

**Figure 7:**
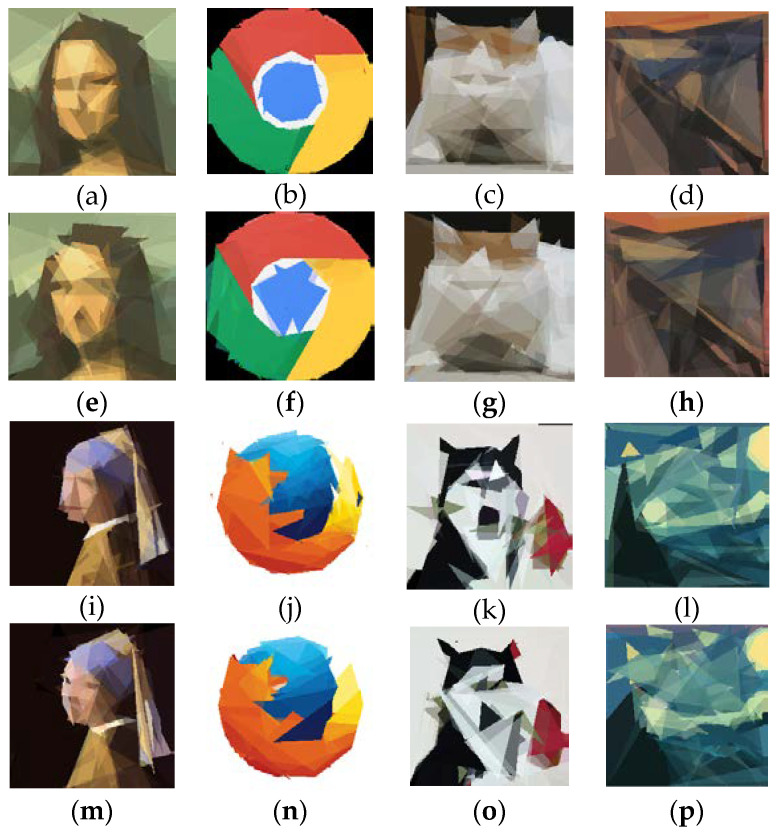
Reconstructed images produced by the two algorithms with 50 triangles. Images (**a**–**d**) and (**i**–**l**) were reconstructed by ProHC-EM. Images (**e**–**h**) and (**m**–**p**) were reconstructed by HC.

**Figure 8:**
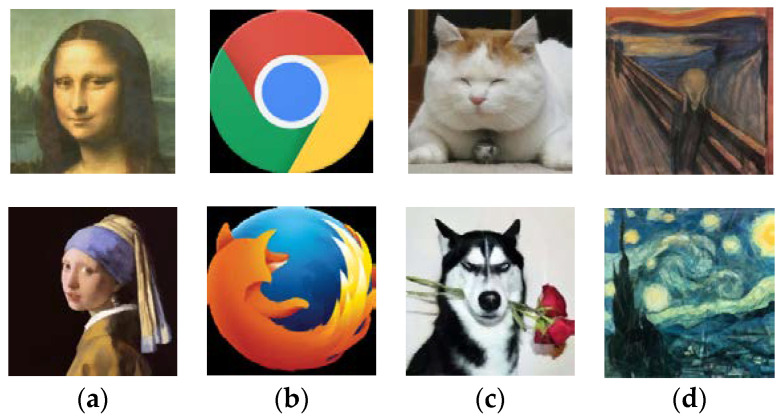
Reconstructed images produced by ProHC-EM with 1000 triangles for the four categories of test cases. (**a**) Reconstructed images of famous portrait paintings; (**b**) reconstructed images of popular browser logos; (**c**) reconstructed images of animal pictures; (**d**) reconstructed images of impressionist paintings.

Figure 7 shows the final results of ProHC-EM and HC for the eight test cases. As can be seen in Figure 7, although the outlines of the reconstructed images were similar to the source images, they were still sketchy. This was because only a small number of polygons (triangles) were used for reconstruction. It was very difficult to capture the details of the source images. To examine the capability of ProHC-EM to fit the images at a fine-grain level, we increased the number of polygons to 1000, and *MaxFEs* was set to 2 × 10^7^. When *m* was set to 1000, there were 10,000 decision variables in total. The hyperparameter *MaxFEs* was set to 1000 times the number of decision variables, enlarged by an additional factor of two. This was because the problem complexity increased dramatically as the number of variables increased. The *CP* indexes achieved by ProHC-EM are reported in Table 2, and the final outputs are displayed in Figure 8. From the figure, it can be seen that more details could be encoded by the parameters of the polygons as the number limit increased.

### 5.3. Running Time Comparison

In this subsection, we compare the running time of ProHC-EM with that of HC. The algorithms were implemented in C++ and compiled using the Microsoft compiler. To calculate the objective function value of a candidate solution, a decoding process that transformed the parameters of the polygons into a reconstructed image was required. The decoding process was implemented using OpenCV functions. Both algorithms were executed on a workstation running Windows 10. The workstation was powered by two Intel Xeon Gold 5218R CPUs with 64 GB memory.

**Figure 9:**
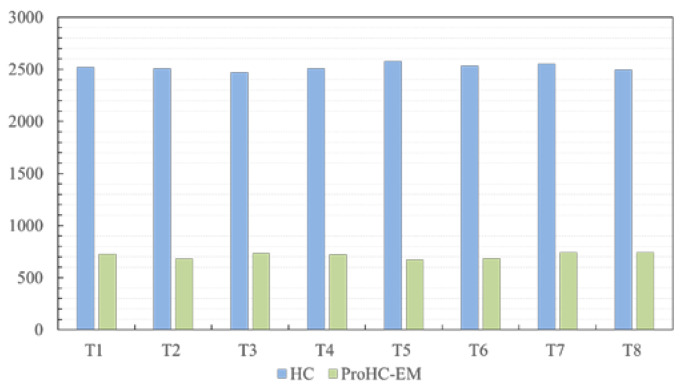
Average running time of ProHC-EM and HC over 25 independent runs for the eight test cases. The *y*-axis shows the running time of the algorithms in seconds.

The experimental results of the algorithms are depicted in Figure 9. The results were averaged over 25 independent runs. From the figure, it can be seen that the average running time of ProHC-EM was much lower than that of HC. ProHC-EM reduced the running time by approximately 60%. This was attributed to the working principle of the progressive learning strategy. Starting from the blank canvas, ProHC-EM stacked the polygons sequentially as the optimization progresses. Therefore, in the early optimization stage, only a small number of polygons needed to be drawn when evaluating the objective function value of candidate solutions. This feature reduced the number of OpenCV function uses. Note that implementing the OpenCV function was the most time-consuming step in the objective function evaluation, so a significant amount of time was saved.

### 5.4. Effect of Mutation Range

In this subsection, we study the effect of the mutation range. The goal of the mutation operator was to find moving directions that could reduce the objective function value by making small changes to the candidate solution. A large mutation range would bring large changes to the candidate solution and cause oscillations, which would be detrimental to the convergence of the algorithm. In Algorithm 3, a random value within 10% of the parameter domain was added to the positional parameters of the polygons. To investigate the influence of the mutation range, nine different settings of the mutation range (i.e., 0.1 to 0.9) were examined on the test cases. The experimental results of ProHC-EM with different mutation ranges are listed in Table 3. It can be observed from the table that the final *CP* indexes dropped as the mutation range increased, indicating that a large mutation range was not suitable for fine-tuning the parameters of the polygons.

### 5.5. Effect of the Energy-Map-Based Initialization

In this subsection, we proceed to study the effect of the energy-map-based initialization operator. To investigate the pure effect of the operator, we removed the operator from ProHC-EM, and the resulting algorithm is denoted as ProHC. ProHC was compared with ProHC-EM to show the influence of the initialization operator on the final solution quality. The experimental results are tabulated in Table 4, with better results highlighted in bold. A Wilcoxon signed rank test was conducted to check whether there were significant differences between the results of ProHC and ProHC-EM. Table 4 also lists the *p*-values output by the statistical tests.

From the table, it can be observed that ProHC was consistently beaten by ProHC-EM in all eight test cases. The *p*-values for all the test cases were smaller than 0.05, indicating that there were significant differences between the results of the two algorithms. With the assistance of the energy-map-based initialization operator, the newly added polygons could be placed on high-energy regions with a high probability. ProHC-EM was capable of reducing the energy by focusing its attention on the most critical region. In this way, the search efficiency was significantly increased. According to the standard deviations listed in the table, the initialization operator could also make the performance of the algorithm more stable.

**Figure 10:**
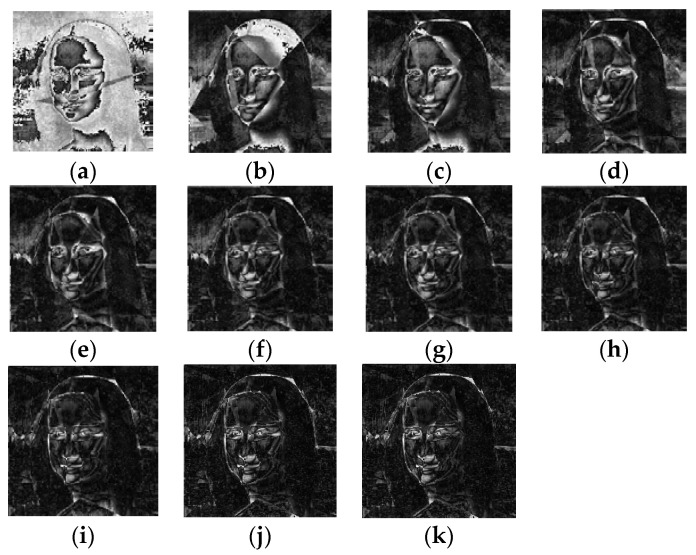
Energy map constructed during the search process of ProHC-EM when solving the first test case: (**a**) *k* = 1, (**b**) *k* = 5, (**c**) *k* = 10, (**d**) *k* = 15, (**e**) *k* = 20, (**f**) *k* = 25, (**g**) *k* = 30, (**h**) *k* = 35, (**i**) *k* = 40, (**j**) *k* = 45, (**k**) *k* = 49.

One thing worth noting is that the energy map had the same size as the source image. The elements in the energy map could be interpreted as pixel values. Therefore, we could display the energy maps as if they were grey images. To demonstrate the changes in the energy maps, Figure 10 depicts the energy maps at different stages of the optimization process when solving the first test case. The brighter the pixel, the higher the energy. It can be observed that at the initial optimization stage, the reconstructed image approximated the source image very poorly. As the optimization progressed, the energy map became darker and darker, indicating that the differences between the reconstructed image and the source image were shrinking. At the end, only small parts of the image had not been recovered by the polygons.

## 6. Conclusions

In this paper, we proposed a progressive learning hill climbing algorithm with an energy-map-based initialization operator to solve the image reconstruction problem. The image reconstruction problem is an interesting yet challenging problem that involves the reconstruction of images using simple geometric shapes. The problem comprises a large number of decision variables that specify the position, color, and transparency of the geometric shapes. The decision variables are highly correlated with each other. To tackle the challenges imposed by this problem, we took inspiration from methods in mathematical optimization and deep learning and developed a progressive learning strategy. The strategy transforms the original complex problem into a sequence of simpler problems. The former problems in the sequence contain fewer decision variables and are easier to solve. Solving the sequence of problems one after another provides a good initial solution to the original complex problem. Furthermore, to increase the search efficiency, an energy-map-based initialization operator was devised to provide good initial positions for the newly added geometric shapes in the progressive learning process.

Comprehensive experiments were conducted on a set of benchmark test cases to examine the effect of the progressive learning strategy and the energy-map-based initialization operator. The experimental results revealed that these processes could enhance the final solution quality and reduce the running time.

For future research, it would be interesting to design more efficient algorithms to reconstruct high-resolution images. A promising approach is to divide high-resolution images into small parts and approximate these small parts separately. The benefit of this approach is that the reconstruction processes for all the small parts can be run in parallel. Another research direction worth investigating is how to measure the differences between the reconstructed image and the source image. In our experiment, the sum of the element-wise absolute differences between the constructed image and the original image was used as the objective function. If our goal is to generate visually similar images, some differences that are imperceptible to humans can be omitted. In this scenario, delta-E [48] can probably be adopted to represent the difference between two colors. We believe that in the future, the emergence of an efficient image reconstruction algorithm will probably give rise to a new sort of compression algorithm, i.e., search-based compression algorithms.

## Figures and Tables

**Table 1:** *CP* indexes of HC and ProHC-EM for the eight test cases. The best *CP* value for each test case is marked in bold.

Test Case		HC	ProHC-EM
T1	Avg. *CP*	90.216022	**91.14915**
	Std.	0.291988	0.315745
	*p*-value	6.57 × 10^−9^	NA
T2	Avg. *CP*	82.286052	**83.368967**
	Std.	0.669923	0.664684
	*p*-value	8.86 × 10^−6^	NA
T3	Avg. *CP*	94.812015	**95.880083**
	Std.	0.5838	0.302158
	*p*-value	3.02 × 10^−7^	NA
T4	Avg. *CP*	94.478735	**95.258706**
	Std.	0.337458	0.268194
	*p*-value	1.46 × 10^−8^	NA
T5	Avg. *CP*	90.902941	**91.236053**
	Std.	0.251318	0.3872
	*p*-value	8.46 × 10^−4^	NA
T6	Avg. *CP*	87.388913	**88.161543**
	Std.	0.595359	0.710967
	*p*-value	1.43 × 10^−4^	NA
T7	Avg. *CP*	82.625574	**83.331248**
	Std.	0.293361	0.284199
	*p*-value	5.55 × 10^−8^	NA
T8	Avg. *CP*	74.261942	**74.601508**
	Std.	0.483394	0.389186
	*p*-value	5.53 × 10^−3^	NA

**Table 2:** *CP* indexes of ProHC-EM for the eight test cases when *m* was set to 1000.

Test Case	ProHC-EM
T1	96.137704
T2	94.523666
T3	99.518189
T4	97.861221
T5	97.038022
T6	96.554793
T7	91.590823
T8	85.322624

**Table 3:** *CP* indexes of ProHC with different mutation ranges for the eight test cases. The best *CP* value for each test case is marked in bold.

Test Case	Mutation Range	0.1	0.2	0.3	0.4	0.5	0.6	0.7	0.8	0.9
T1	Avg. *CP*	**91.14915**	90.87189	90.70037	90.59070	90.50501	90.34281	90.48234	90.33044	90.26642
	Std.	0.31575	0.33278	0.35830	0.33569	0.38663	0.35651	0.31498	0.33974	0.41427
T2	Avg. *CP*	**83.36897**	82.47898	82.61518	82.50591	82.22858	81.93730	82.22872	81.25461	81.25461
	Std.	0.66468	0.78430	0.80984	0.96699	0.74271	0.73894	0.88683	0.95879	0.95879
T3	Avg. *CP*	**95.88008**	95.52075	95.49631	95.46927	95.13949	95.06237	94.89285	95.10121	95.23188
	Std.	0.30216	0.51806	0.45107	0.52618	0.65007	0.70568	0.58218	0.51750	0.76543
T4	Avg. *CP*	**95.25871**	92.17570	91.84952	91.73971	91.86117	91.65076	91.43410	91.78058	91.52568
	Std.	0.26819	0.63846	0.56765	0.75720	0.63565	0.56570	0.71608	0.54735	0.52018
T5	Avg. *CP*	**91.23605**	89.41844	89.11147	88.81204	88.72750	88.76321	88.81424	88.66988	88.55271
	Std.	0.38720	0.58290	0.56171	0.55275	0.57020	0.56621	0.56117	0.66138	0.54113
T6	Avg. *CP*	**88.16154**	83.04914	82.36449	82.21335	82.22623	82.46466	81.59485	81.74754	81.67813
	Std.	0.71097	0.89787	1.09454	0.86865	0.89170	0.80457	0.81495	0.62258	0.67681
T7	Avg. *CP*	**83.33125**	83.29202	83.06892	82.87703	83.01335	83.00752	82.91494	82.87398	82.65907
	Std.	0.28420	0.32702	0.41808	0.43390	0.46488	0.43877	0.31222	0.33542	0.51470
T8	Avg. *CP*	**74.60151**	74.41921	74.25212	74.14972	74.05667	74.25279	73.88122	73.86801	74.00551
	Std.	0.38919	0.48475	0.55026	0.58210	0.51648	0.48708	0.59222	0.46899	0.50670

**Table 4:** *CP* indexes of ProHC and ProHC-EM for the eight test cases. The best *CP* value for each test case is marked in bold.

Test Case		ProHC	ProHC-EM
T1	Avg. *CP*	90.395986	**91.14915**
	Std.	0.708987	**0.315745**
	*p*-value	5.91 × 10^−5^	NA
T2	Avg. *CP*	81.990398	**83.368967**
	Std.	1.197063	**0.664684**
	*p*-value	4.24 × 10^−5^	NA
T3	Avg. *CP*	94.300562	**95.880083**
	Std.	1.066847	**0.302158**
	*p*-value	5.85 × 10^−9^	NA
T4	Avg. *CP*	94.407076	**95.258706**
	Std.	0.668713	**0.268194**
	*p*-value	3.53 × 10^−6^	NA
T5	Avg. *CP*	90.270867	**91.236053**
	Std.	0.580166	**0.3872**
	*p*-value	4.10 × 10^−7^	NA
T6	Avg. *CP*	87.462471	**88.161543**
	Std.	0.947679	**0.710967**
	*p*-value	5.21 × 10^−3^	NA
T7	Avg. *CP*	82.71647	**83.331248**
	Std.	0.506035	**0.284199**
	*p*-value	8.10 × 10^−6^	NA
T8	Avg. *CP*	73.668609	**74.601508**
	Std.	0.746615	**0.389186**
	*p*-value	2.92 × 10^−6^	NA

## Data Availability

The data presented in this study are available on request from the corresponding author.
